# MR imaging features of orbital Langerhans cell Histiocytosis

**DOI:** 10.1186/s12886-019-1269-9

**Published:** 2019-12-19

**Authors:** Chunnan Wu, Kuncheng Li, Yan Hei, Pengyu Lan, Xuetao Mu

**Affiliations:** 1 Department of MRI, The Third Medical Center of Chinese PLA General Hospital, Beijing, 100039 China; 2 Department of Radiology, Xuanwu Hospital, Capital Medical University, Beijing, 100053 China; 3 Department of Pathology, The Third Medical Center of Chinese PLA General Hospital, Beijing, 100039 China; 4 Department of CT, The Third Medical Center of Chinese PLA General Hospital, Beijing, 100039 China

**Keywords:** Orbit, Langerhans cell histiocytosis, MRI features

## Abstract

**Background:**

To investigate the magnetic resonance imaging (MRI) features of orbital Langerhans cell histiocytosis (LCH) to improve diagnostic accuracy.

**Methods:**

We retrospectively reviewed clinical manifestations and MRI findings of 23 patients with histopathology-confirmed LCH of the orbit. The findings were evaluated for the following: (a) symptoms, (b) disease duration, (c) location, (d) configuration, (e) margin, (f) MR imaging signal intensity and enhanced performance.

**Results:**

Eighteen patients (78%) in our series were male, only five (22%) patients were female, and the mean age at presentation was 6.3 years. The common symptoms include swollen eyelids, exophthalmos, and a palpable mass. Fourteen patients presented with swollen eyelids and/or exophthalmos. Twenty-two cases involved unilateral orbits, and one case involved bilateral orbits. In our study, there was one patient with cough and expectoration, and one patient with polydipsia and polyuria. Lesions were located in the superior or superlateral orbital roof of seventeen patients (74%). Lesions formed masses or irregular shapes. The 12 out of 23 (52.2%) cases appeared heterogeneous isointense and 10 out of 23 (43.5%) cases showed iso-hypointense on T1-weighted imaging, there were 15 out of 23 (65.2%) cases showed hyper-hypointense mixed signals on T2-weighted imaging. 7 cases found patchy hyperintense signal on T1WI, and 11 cases showed markedly hyperintense signal near the edge of lesions on T2WI. After enhancement, 21 out of 23 (91.3%) cases lesions presented marked enhancement at the edges and the surrounding tissues, and with heterogeneous obvious enhancement of the lesion center. Besides, four cases lesions were surrounded by a low circular signal.

**Conclusion:**

There were several characteristics MRI features that can provide crucial information for clinicians and improve our understanding and the diagnostic accuracy of the orbital LCH.

## Background

Langerhans cell histiocytosis (LCH) is a rare disease of the dendritic cell system that may occur at any age, with a peak incidence between 5 and 10 years, so it has a high incidence in children, and it often involves the orbit [1–3]. Its histopathologic landmark is a granulomatous infiltrate of histiocytes of Langerhans cell phenotype (CD1a+), interspersed with varying proportions of macrophages, T-lymphocytes, eosinophils and multinucleated giant cells. The pathogenesis of LCH had not been fully clarified. The demonstration that lesional Langerhans cell are clonal, LCH is regarded as a reactive clonal disease of the monocyte-macrophage system and may affect almost any organ. Clinical presentation and course of LCH are widely heterogeneous and may rang from self-healing isolated skin or bone lesion(s), to disseminated disease with potentially lethal tissue damage [4], so it is recognized as a spectrum of diseases [5, 6].

In rare instances, the affection of the orbit is the only and the first symptom. Orbital LCH is a rare clinical entity and presents a diagnostic dilemma because of its radiological features. Diagnosis of LCH is challenging as it may simulate periorbital hematoma, rhabdomyosarcoma, and neuroblastoma malignant lacrimal gland tumor, orbital cysticercosis, and orbital tuberculosis [7]. In addition, there was currently little evidence that unifocal orbital disease increases the risk for CNS-LCH and CNS-LCH would affected the quality of patient’s life obviously [8]. Therefore, a detailed research is required to find the radiological features to improve the diagnosis of the orbital LCH.

In the previous literature, there were some reports about the magnetic resonance imaging (MRI) feature of CNS LCH, or LCH in the pelvis and extremities those extraorbital lesions [4, 9, 10]. As for the orbital LCH, literature mostly reported on the treatment in the form of case reports, or the number of cases was less than ten with imaging data being limited [3, 11]. To the best of our knowledge, we didn’t find the studies focused on MRI performance of orbital LCH.

Herein, we analyzed the clinical features and MRI characteristic manifestations of 23 patients with orbital LCH in detail. Our aim is to find the characteristic features in MRI, and improve our understanding of the disease and the diagnostic accuracy.

## Methods

### Patients

In our retrospective study, we collected 23 patients with histopathology-confirmed LCH of the orbit from 2008 to 2016. Seventeen of the patients were males and five female. Their ages at diagnosis of LCH ranged from 9 month to 21 years. The inclusion criteria were: (a) histopathologically confirmed cases of LCH; (b) patients with orbital MRI (including pre- and post-contrast studies) less than seven days before biopsy or surgery; (b) patients with no history of surgery or treatment in the affected orbits. The exclusion criteria were as follow: (a) poor image quality such as significant motion or susceptibility artifacts; (b) orbital lesions less than 0.5 cm in diameter; (c) patients with orbital LCH recurrence after surgery. All participants involved were informed of the purpose of this study and a written consent was obtained from themselves or their parents or legal guardians.

#### Hematoxylin-eosin (HE) and Immunohistochemical (IHC) staining

HE staining was conducted according to routine protocols [12]. Excised post-surgery material specimens were embedded in paraffin and cut into 4 μm sections. Sections were then deparaffinized by xylene followed by decreasing concentration of ethanol (100–50%) and finally washed in cold tap water. Endogenous peroxidase activity was neutralized by incubation with 0.5% hydrogen peroxide in methanol. Antigen retrieval used high temperature-pressure method in pH 8 EDTA buffer. Following antigen retrieval, put in the 3% hydrogen peroxide solution for 5–10 min, remove the endogenous peroxidase, and then put in PBS buffer 3 min to wash three times. Samples were incubated with primary antibody to S-100 (Biocare, U.K., ZM-0224, 1:150); CD 68 (Zeta, US, ZM-0060, 1:200) and CD1a (Epitomics, US, ZA-0544, 1:200). Following primary antibody incubation slides were washed and secondary antibody PV-6000 (Beijing Zhongshan Golden Bridge Biotechnology Co. LTD, Beijing, China) was applied. After another washing, DAB (3,3’Diaminobenzidine) substrate was applied. Slides were counterstained with hematoxylin.

#### Image data acquisiton

All MRI examinations were performed on a 3.0 T MR imaging system (Trio Tim Siemens, Germany) with an 8-channel head coil. Pre-contrast axial fast spin-echo (FSE) T1-weighted imaging (T1WI), T2-weighted imaging (T2WI) in the axial and coronal planes and post-contrast T1WI in the axial, coronal, and sagittal planes were acquired in all cases. The imaging parameters were as follows. T1WI: TR, 200 ms; TE, 2.46 ms; Angle, 70 °. T2WI: TR, 3400 ms; TE, 108 ms; Angle, 120°; vision, 190 mm × 190 mm; matrix, 256 × 256; section thickness, 3 mm; intersection gap, 0.3 mm. Post-contrast T1WI were obtained after an intravenous bolus injection of 0.2 ml/kg of Gd-DTPA (Gadopentetate Dimeglumine Injection; Consun pharmaceutical co., Guang zhou, China). Chemical shift selective fat saturation (FS) was used in the post-enhancement axial T1WI.

### Medical record and MRI imaging analysis

Medical records were analyzed for age at presentation, gender, laterality, symptoms and the disease course. The MR images were transferred to a picture archiving and communicating system (PACS), then the images reviewed by two head and neck radiologists. The analysis of lesions included: location, morphology, size, boundary and pre- and post-contrast MRI performances. If they did not agree on a diagnosis, then another imaging doctor provided a common consensus diagnosis through consultation.

## Results

### Clinical characteristics of the patients

Eighteen patients (78%) in our series were male, only five (22%) patients were female, and the mean age at presentation was 6.3 years. Their most common symptoms included eyelid swelling, pain, exophthalmos and local masses. Twenty-two cases had unilateral disease with left eye being involved in ten and right in twelve patients, and only one case had bilateral orbit LCH. All patients had short disease duration from six days to one year, 21 cases occurring within 2 months. The majority patients had different degrees of eyelid swelling. Fourteen patients had red swollen eyelids and exophthalmos; Among them, six cases showed recurrent eyelid swelling, pain and fever, which like the symptom of inflammatory pscudotumor, the anti-inflammatory treatment was ineffective; two patients had ptosis; two patients had throbbing sensation, and one patients accompanied by tearing, one accompanied by cough and sputum, one accompanied by polydipsia polyuria, One patient had eyelid swelling after trauma. Four patients only had a swollen eyelid with no redness or pain. Three patients only presented with a palpable mass at periorbital with no other clinical manifestations. Two patients had temporal pain accompanied by blurred vision, one of them had nausea and vomiting, the other had dizziness and tinnitus. In our cases, there were two patients have slight vision decline (visual chart: 0.8, 0.7), and the other patients’ vision were normal. Due to the lesions of LCH were not affect the optic nerve or orbital apex, 21 out of 23 cases (91.3%) patients’ vision is unaffected.

**Fig. 1:**
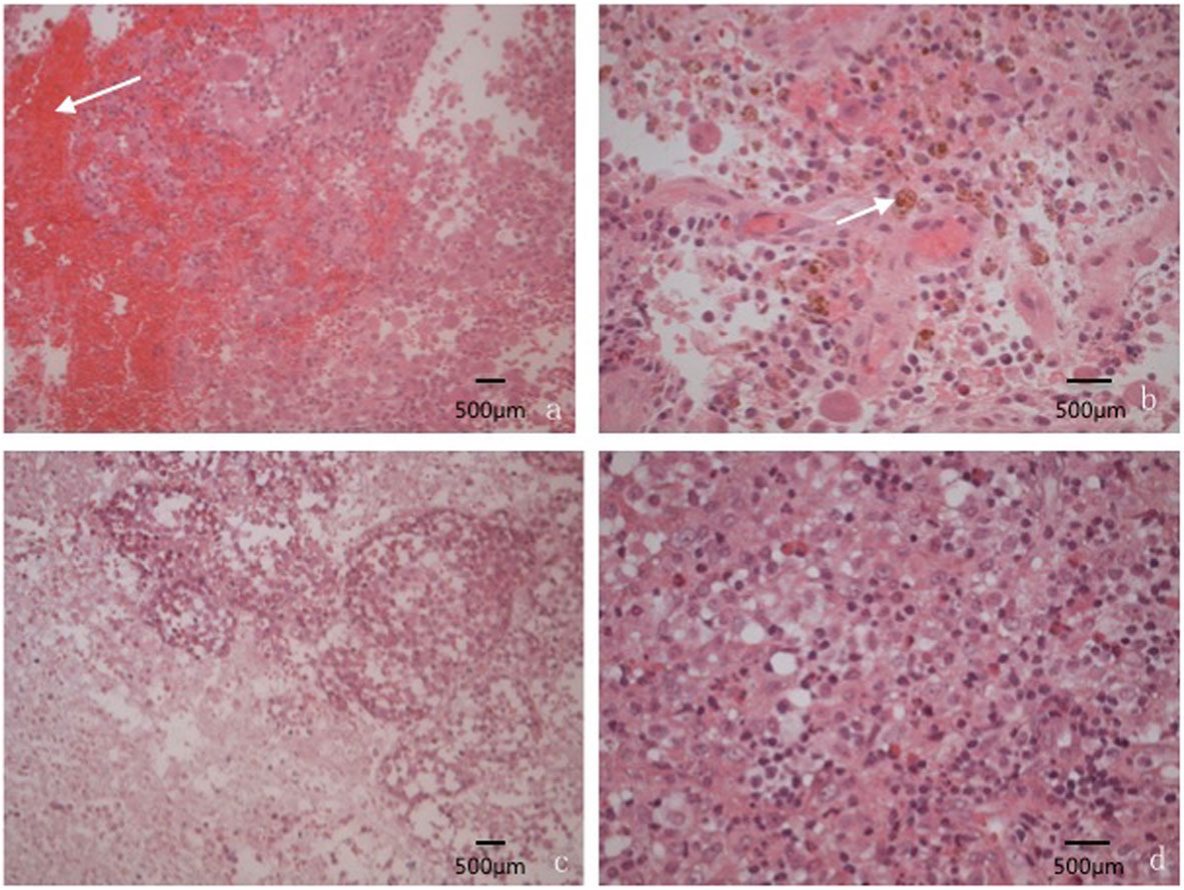
Representative image of HE stain (**a**): Dense infiltrate of Langerhans-type histiocytes and eosinophilic cells with many red blood cells (× 200). (**b**): A large amount of hemosiderin and macrophages which contained hemosiderin(× 400). (**c**): A large amount of necrotic tissue within the tumor cells(× 200); (**d**): A large proliferation of Langerhans cells, eosinophils and neutrophils as well as lymphocytes with scattered plasma cells and blood cells(× 400)

**Fig. 2:**
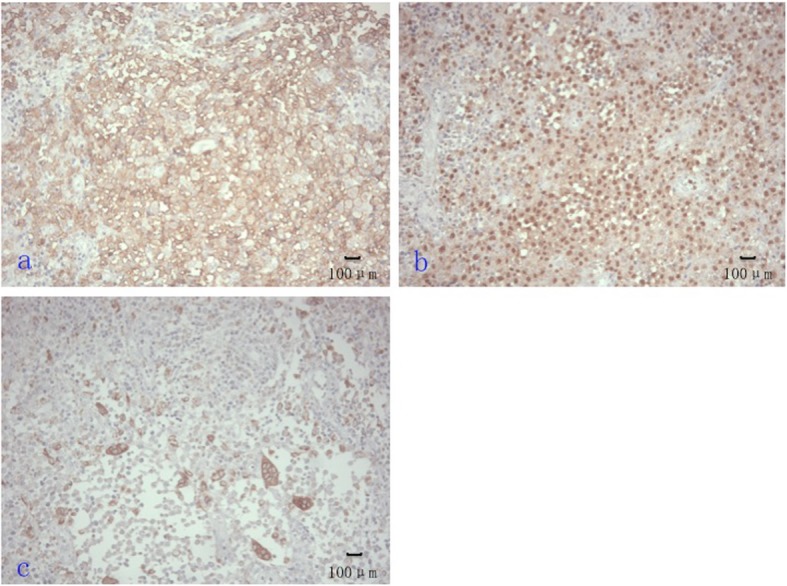
Representative images of IHC stain: (**a**): CD 1a positive (× 200), (**b**): S100 positive (× 200), (**c**): CD68 positive (× 200)

HE stains showed that the lesions include the proliferation of large number of Langerhans cells with eosinophils and neutrophils, lymphocytes and a small amount of plasma cell infiltration. The IHC stains shows positive signals for S – 100, CD 68 and CD1a (Fig. 1, 2).

### Location and morphology of lesions

**Fig. 3:**
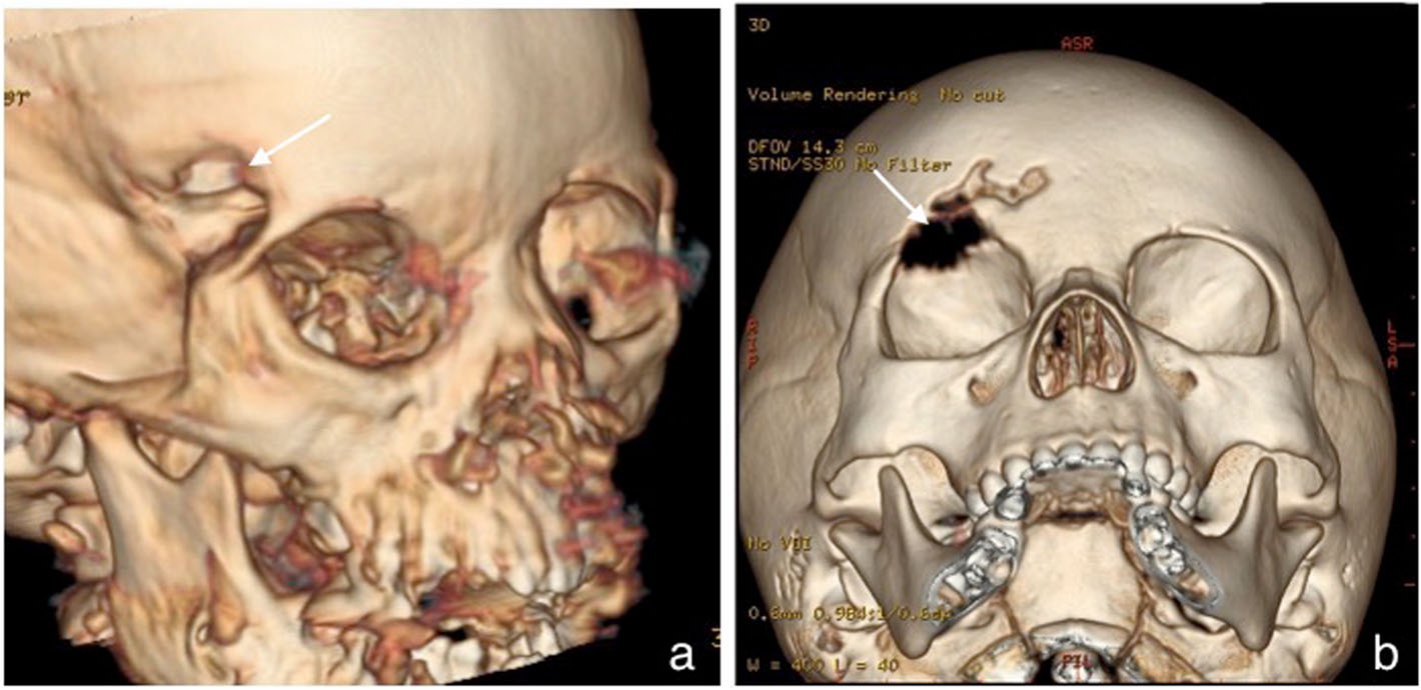
Representative 3D reconstructions on CT data: it can show the lesion site very well. a: lesion located the superlateral roof of the right orbit, b: lesion located the superior roof of the right orbit

**Fig. 4:**
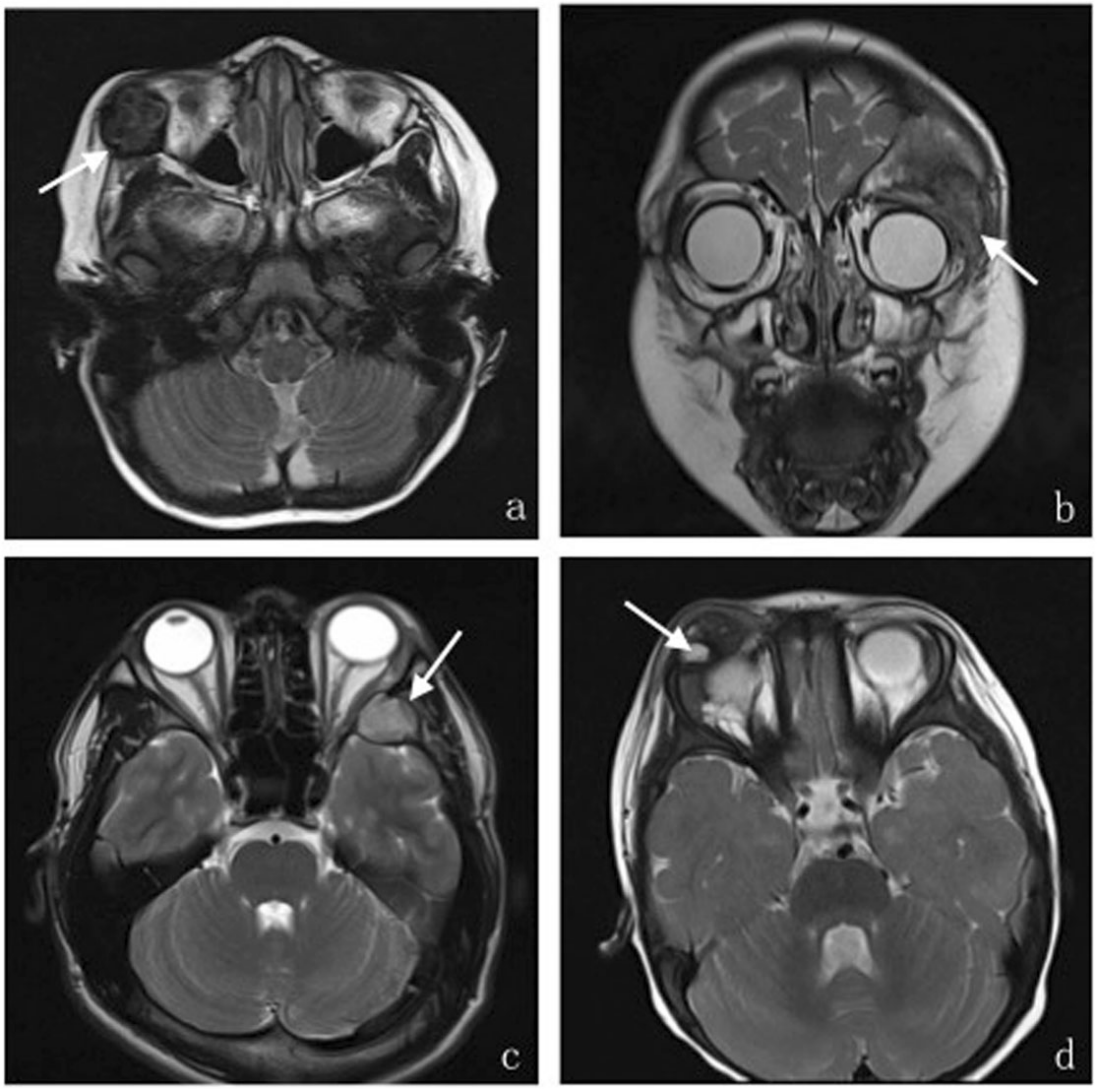
**a**–**d**. The different performances of orbital LCH lesions in T2WI:Axial (**a**) T2-weighted MR images show lesion located in the inferolateral right orbit with a clear border, and presenting hypointensity in a T2-weighted image with a low signal ring of the lesion edge;and coronal (**b**) T2-weighted MR images show lesion located in the right superolateral orbit with an irregular shape like a triangle and an unclear boundary, presenting hyper-hypointense mixed signals; Axial (**c**) T2WI show lesion located in the lateral wall of the left orbit presenting slightly hyperintense signals with a clear boundary. Axial (**d**) T2WI show lesion located in the superior wall of the right orbit presenting multiple cystic hyperintense signals near the edge of the lesion

**Fig. 5:**
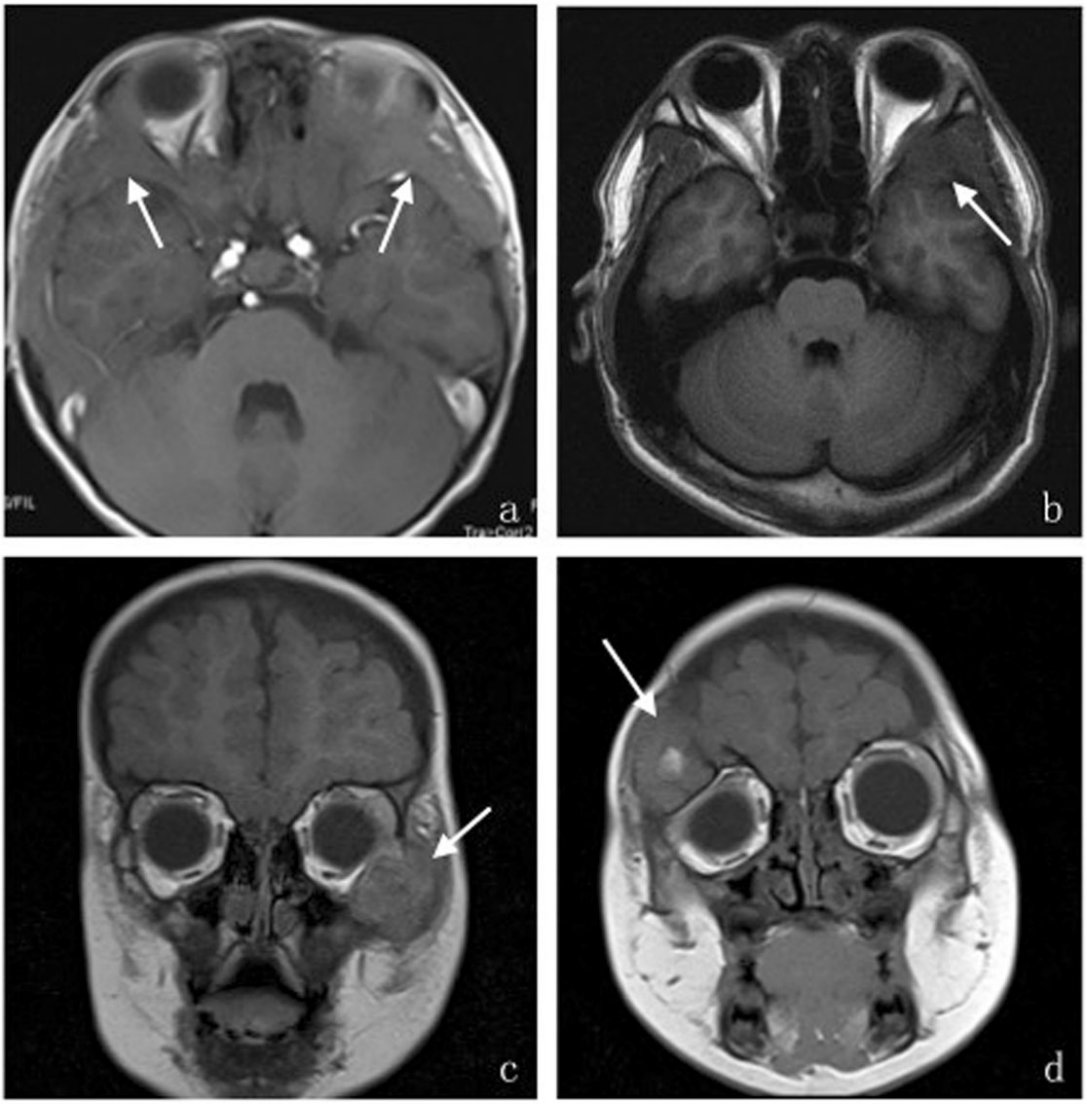
**a**–**d**. The different performances in T1WI.Axial (**a**) T1-weighted MR images show isointensity, and the lesion performs diffuse thickening of the affected bone with ill-defined margins; Axial (**b**) T1WI show lesion located in the left lateral orbital wall presenting hypointensity and an unclear boundary; Coronal (**c**) T1-weighted MR images show mass located in the inferolateral left orbit presenting iso-hypointense mixed signals and an unclear boundary. Coronal (**d**) T1WI show lesion located in the superolateral right orbit performing isointensity with patchy hyperintensity inside

There were 13 lesions located in the superolateral orbit, 5 lesions located in the lateral orbit, 3 lesions located in the inferolateral orbit, 1 lesion located in the orbital apex, and 1 case of bilateral orbit lesions. All lesions involved the orbital wall. We can clearly see the bone destruction on the 3D reconstructions of CT data (Fig. 3), and it can show the lesions site very well. In all, 4 involved the extra-orbital bone skull included tibia, foot bone, mandibular, cheekbones and sphenoid bone. The size of the largest lesion was 2.2 cm × 4.0 cm × 5.4 cm, and the smallest was 0.9 cmx 1.0 cmx 1.0 cm. Fourteen cases appeared as a mass or an ovoid configuration (Fig. 4a), 7 cases had an irregular shape like a triangle (Fig. 4b), and the other two cases had diffuse thickening of the affected bone with ill-defined margins (Fig. 5a). Fifteen cases had unclearly boundary, and 8 cases had clear boundary. (Table 1).

**Table 1:** The location, shape and boundary of orbital LCH

location		morphology		boundary	
positon	case number	shape	case number		
superlateral	13	mass	14	unclear	15
lateral	5	triangular	7	clear	8
inferolateral	3	diffuse thciken	2		
orbital apex	1				
orbital lateral	1				

### Pre-contrast MRI presentation along with the pathological results (Table 2)

T2WI performance (Fig. 4): 15 cases showed hyper-hypointense mixed signals on T2WI, 5 cases showed slightly hyperintense signals, and the other 3 cases showed hypointense signals primarily, the HE stain image showed large amounts of hemosiderin and macrophages which phagocytosed hemosiderin under the microscope (Fig. 1a, b). Among all cases, 11 cases showed cystic hyperintense signal near the edge of lesions on T2WI.

**Table 2:** The T2WI, T1WI and enhancement signal performance of orbital LCH

T2WI	T1WI	Enhancement signal	Case No.
hyper-hypointense mixed signal	isointensity	significantly heterogeneous	5
hyper-hypointense mixed signal	iso-hypointensity	significantly heterogeneous	4
hyper-hypointense mixed signal	isointensity	significantly heterogeneous	1
hyper-hypointense mixed signal	isointensity	flocculent\septations	1
hyper-hypointense mixed signal	iso-hypointensity	flocculent\septations	2
hyper-hypointense mixed signal	iso-hypointensity	slight or moderate heterogenous	2
slight hyperintensity	isointensity	slight or moderate heterogenous	1
slight hyperintensity	iso-hypointensity	slight or moderate heterogenous	1
slight hyperintensity	isointensity	slight homogenous	1
slight hyperintensity	hypointensity	slight homogenous	1
slight hyperintensity	iso-hypointensity	homogenous enhancement of thickening bone	1
hypointensity	isointensity	homogenous enhancement of thickening bone	1
hypointensity	isointensity	significantly heterogeneous	2

Note: besides two cases showed homogenous enhancement of thickening bone, other 21 cases presented significantly enhancement at the edges of lesions and surrounding tissues around the lesion

T1WI performance (Fig. 5): 12 cases showed isointensity on T1WI, 10 cases showed iso-hypointense mixed signals, and one case showed a hypointense signal. Among all cases, patchy hyperintense signals were found in 7 lesions on T1WI, where many red blood cells and hemosiderin were seen under the optical microscope (Fig. 1a, b), according to the focal hemorrhage. For both T1WI and T2WI, only 3 lesions showed homogeneous signals, 4 lesions were surrounded by a ring of hypointense signal.

### Post-contrast MRI presentation

**Fig. 6:**
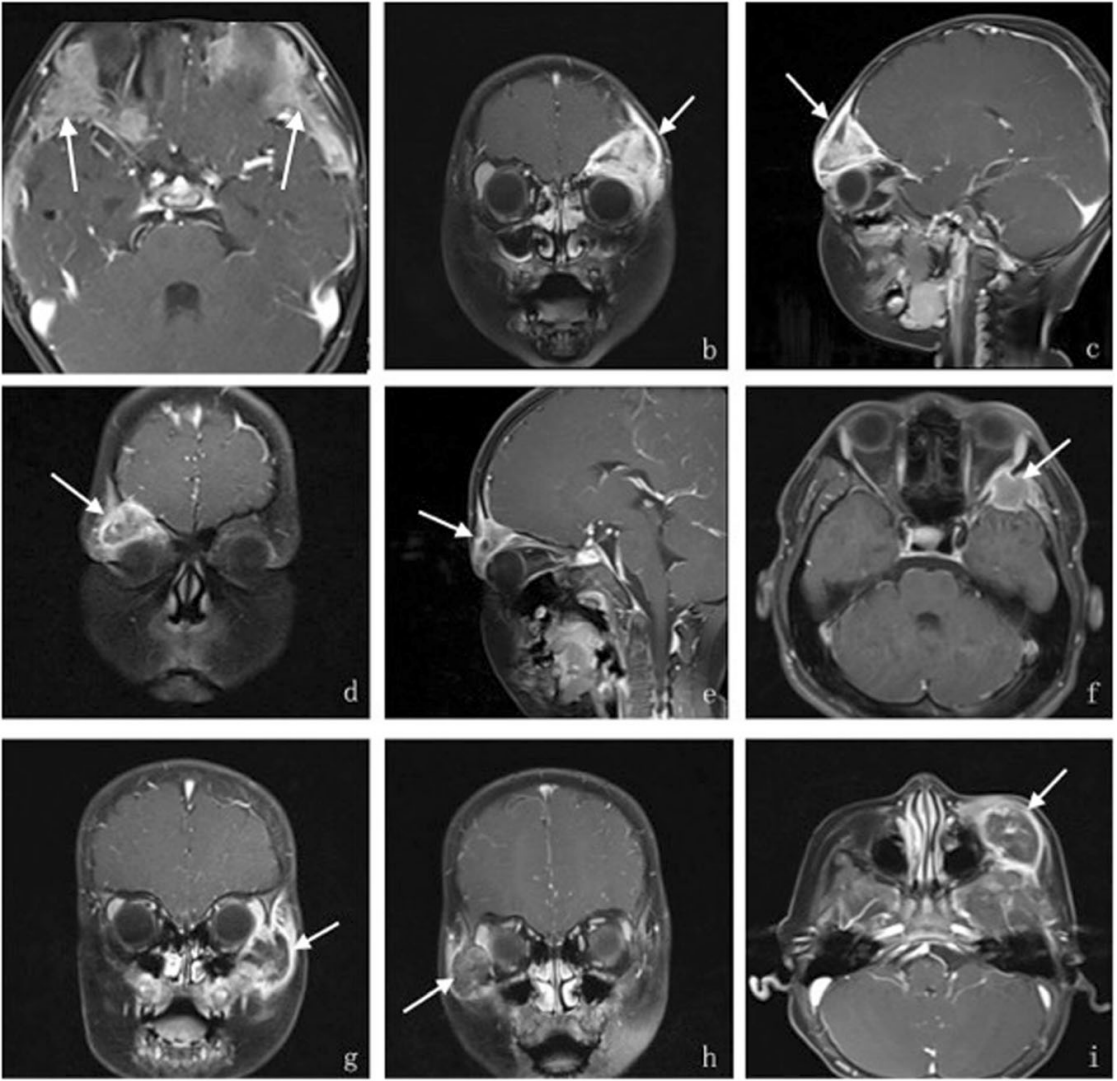
a-i. The different performances of orbital LCH lesions in enhanced T1-weighted images. Axial T1-weighted postcontrast images (**a**) demonstrate homogeneous enhancement; Coronal (**b**) and sagittal(**c**) T1-weighted postcontrast images represent the same patient with a lesion located in the superolateral of the left orbit, the lesion shaped like a triangle crumb presenting significant heterogeneous enhancement with some irregular low signal areas near the lesion edge. Coronal (**d**) and sagittal(**e**) T1-weighted postcontrast images represent the same patient, the lesion is located in the superolateral right orbit with an irregular triangle shape, an unclear boundary, and slight or moderate heterogeneous enhancement. Axial T1-weighted postcontrast images (**f**) demonstrate a lesion located in the lateral wall of left orbit and shows slight homogeneous enhancement around the center of the lesion. Axial (**g**) and Coronal (**h**) T1-weighted postcontrast images represent the same patient with a lesion showing flocculent enhancement in the central hypointense area, and the area surrounding the lesion showed more significant enhancement. Coronal (**i**) T1-weighted postcontrast images showing slight-moderate heterogeneous enhancement in the center of the lesion, and surrounded by a circular low signal

Contrast-enhanced image performance: There were 2 cases presented bone thickening and showed homogeneous enhancement (Fig. 6a), the other 21 cases presented significantly enhancement at the edges of lesions and surrounding tissues around the lesion (Fig. 6). At the center of the lesions, 12 cases showed significantly heterogeneous enhancement (Fig. 6 b, c), internal no enhancement area was consistent with necrotic tissue in the HE stain image (Fig. 1c). 4 cases showed slight or moderate heterogeneous enhancement (Fig. 6 d, e), 2 cases showed slight homogeneous enhancement (Fig. 6f), and the other 3 cases showed flocculent or septations enhancement in the hypointense central area (Fig. 6g, h) In addition, there were 4 cases of lesions surrounded by a low circular signal (Fig. 6i).

## Discussion

### Summary of Langerhans cell histiocytosis

LCH includes three syndromes: eosinophilic granuloma in bone, Hand - Schüller - Christian disease and Letterer - Siwe disease. According to the degree of clinical damage, LCH can be divided into a localized type and an extensive type. Among the three syndrome types, bone eosinophilic granuloma is a localized type, Hand-Schüller-Christian disease progresses slowly and is likely an extensive type, and Letterer-Siwe disease progresses quickly and urgently, which likely makes it an extensive type with a poor prognosis [13]. LCH can involve multi-organ and multisystem. Lung and bone were the more frequent involved regions. For the single bone destruction, can be simple surgical curettage. In case of the recurrence patient may require systemic chemotherapy. According to patient’s concrete condition, arrange periodic reviews from 6 weeks to one year. In our group, all 23 cases underwent surgical resection, and postoperative chemotherapy was given subsequently, most of the cases have a good prognosis up to now.

### The clinical manifestations of LCH with orbital involvement

**Fig. 7:**
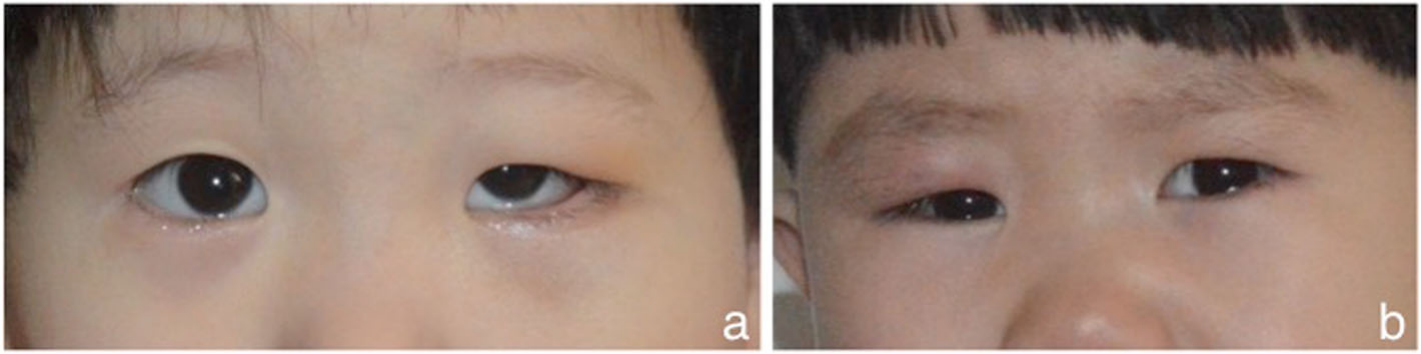
Representative photographs: Photographs of two child patients’ to present the eyes overall look. (**a**): eyelid swelling of left orbit; (**b**): red swollen eyelid of right orbit.

LCH is a rare disease with an incidence of 0.2–2.0 cases per 100,000 children under 15 years of age in the worldwide [13], and it has a high incidence 23–37.5% of orbital involvement [14, 15]. In our study, the average age was 6.3 years, including 20 patients (86.9%) under the age of five, and 2 cases occurred in adults who were 21 years old and one patient was 14 years old. There were 18 male patients (78.3%). The results demonstrated that LCH are more common in pre-adolescent males, that is the same with the previous literature [16]. From patients recognizing the symptoms to the diagnosis, the course of disease were shorter, there were 15 cases that lasted less than one month and 5 cases that lasted approximately two months. The most common clinical manifestations included eyelid swelling and exophthalmos. Majority patients had swollen eyelids (Fig. 7), including 14 cases accompanied with pain and fever and 6 cases with poor treatment effects after a mistaken diagnosis of an inflammatory pseudotumor, this led us to the conclusion that the clinical manifestations of orbital LCH were almost similar to the inflammatory pseudotumor, and it is apt to be misdiagnosed. In our research group, patients also had some relatively rare symptom: ptosis, fluctuations in the affected part, tearing, decreased vision, dizziness, and nausea clinical symptoms which have not been mentioned in previous studies [3, 5, 17]. There was one child appeared lump after trauma, a previous literature reported that trauma might be a factor that can trigger an immune response, and predisposed children may subsequently develop LCH [18]. Three patients were diagnosed with eosinophilic granuloma of the bone, and one case presented “bone destruction-exophthalmos-diabetes insipidus” syndrome, which is the trigeminy sickness of the Hand-Schüller-Christian disease. Regrettably, due to a lack of body multi-system check data, we could not determine the exact type of the remaining cases.

### Magnetic resonance imaging findings of orbital LCH

MRI has a high resolution of soft tissue, and can clearly show the skull bone destruction, soft tissue mass, dural thickening and other morphological changes of LCH lesion in orbit, but the MRI signal of LCH lesions changes in a large range, the MRI features reported in the literature are not consistent [19–21]. In fact, according to the results of our study, we found that the MRI manifestation of orbital LCH possess certain characteristics actually. In our group, 17 lesions located in the superior or superlateral orbital roof that accounted for 74% of the total cases. So we know that orbital LCH mainly occurs in the superior or superlateral wall of orbit. It should be noted that the lesions locations were superficial. We found that mostly lesions of orbital LCH presented as a mass or triangular, the triangular lesions were similar in shape to the bone section of the site, and 3 cases showed bone thickening. From this representation, we speculate that the shape of the first-stage lesion is the same as the original bone form, and then it further progressed to a lump shape. In our group, most lesion boundaries were not clear except for 3 cases, and those 3 cases were diagnosed as bone eosinophilic granuloma by pathology, the characteristics was consistent with the findings of previous study [22]. The boundaries of other types of LCH lesions are often unclear.

Based on our MRI observations, most lesions appeared isointensity and iso-hypointensity on T1WI. On T2WI, the lesions mostly exhibited hyper-hypointense mixed signals. Most lesions presented heterogeneous signal, some studies considered that heterogeneous signals are related to different pathological stages or the lipid content of lesions [23, 24]. Histological observations of pathological material revealed, we found that lesions containing many red blood cells, where presented patchy hyperintense signals on T1WI and conformed to a focal hemorrhage. Another feature is that the area near the edge of lesions usually had capsule hyperintense signals on T2WI and which were not enhanced, these areas correlated with the necrosis area of lesions shown by a microscope (Fig. 1, 2). Post enhancements revealed that lesions mostly showed significantly heterogeneous enhancement signals, and we found a key characteristic that the edges of the lesion and surrounding tissue presented significant enhancement, which was more obvious than the central of the lesion. We suggest that this characteristics performance may help in the diagnosis of orbital LCH. Besides, A few lesions were surrounded by a low signal ring on the pre-enhanced and post-enhanced imaging, which did not been reported before, and determining its diagnostic value requires further observation.

Believed through ours research on the MRI feature of the orbital LCH, lesions of LCH were pone to occur at the superior and superlateral wall of orbit, and the locations were superficial that near the surface (Fig. 3). The shape of lesion like lump or triangular with fuzzy boundaries. It presented isointensity and iso-hypointensity on T1WI and hyper-hypointense mixed signals on T2WI, pone to hemorrhage and necrosis in lesions. It need to be emphasized that the edges of the lesion and surrounding tissue presented more significant enhancement, it concluded that LCH lesions affected the surrounding soft tissue. Base on above MRI characteristics performance, it can improve the diagnostic accuracy of the orbital LCH for radiologists. These specific MRI manifestations have not been previously reported.

### The differential diagnosis of orbital LCH

The diagnosis and differential diagnosis of LCH is mainly based on clinical manifestation, imaging and pathological examinations, and imaging examination especially plays an important role in the diagnosis of the disease, but the final diagnosis is still requires histopathology for confirmation. The histopathological features of LCH include the proliferation of large number of Langerhans cells with eosinophils and neutrophils, lymphocytes and a small amount of plasma cell infiltration. The IHC staining shows positive signals for S – 100, CD 68 and CD1a (Fig. 1, 2). “Birbeck granules” can be found under the electron microscope, which is the gold standard for diagnosing LCH. But electron microscope may not be widely available for clinical purposes, the MRI and bioptate IHC findings are most available methods for LCH diagnostics. This signifies the importance of the MRI manifestations as MRI is widely available in clinical practice and that is a non-invasive method of diagnostic.

The main differential diagnoses for orbital LCH include leiomyosarcoma, metastases, chloroma, orbital inflammatory pseudotumor, epidermoid or dermoid cysts, multiple myeloma, et al. (1) Leiomyosarcoma: this tumor is also a common orbital malignancy in teenagers, progresses very quickly, often occurs at the outer upper quadrant of the extraconal orbital compartment, the location of lesions often deeper than the LCH. On MRI, the lesion mostly show low signal on T1WI, high signal on T2WI, and have homogenous and heterogeneous enhancement. It should be pointed out that the lesion is rarely hemorrhage, the edges of the lesion and surrounding tissue are not enhanced, that can be used to differentiated from the orbital LCH. And the lesion often has a little bone destruction. (2) Metastatic tumors: metastases in teenagers often come from neuroblastoma, most neuroblastomas occur in the retroperitoneal and adrenal areas. Patients demonstrate symptoms outside the orbital region, consistent with primary tumor localization, along with typical malignancy associated general symptoms. The lesion progresses fast, most patients had invasive bone destruction , that is different from the bone defect of LCH. Presence of the primary tumor is the main finding in the differential diagnostic of the condition [25]. (3) Chloroma: Leukemia cells infiltrating the orbital bone and soft tissue. The lesion show low signal on T1WI, slight-high signal on T2WI, and have a significantly homogenous enhancement, the MRI features are different from the LCH. The general condition of leukemia patients is poor, and systemic examination can ultimately diagnose leukemia [26]. (4) Epidermoid or dermoid cysts are more common in middle-aged individuals, have a slow onset, and present pressure changes in bone. MRI signals of lesions change and present capsule wall enhancement as well as internal structures without enhancement. (5) Myeloma is more common in middle-old aged individuals; lesion show low signal on T1WI, high signal on T2WI, and have a a significantly homogenous enhancement, and present oval bone destruction on X-ray approximately 50 to 70% of the patients are positive for Bence-Jones protein.

The limitation of the current study is that only some patients from the study group had data available for diffusion-weighted magnetic resonance imaging (DWI) and dynamic enhanced scanning; these results were not included in the current study. However, from the partial datasets available (data not shown), it appears DWI, and dynamic enhanced scanning doesn’t have the characteristic that can be helpful for LCH diagnosis.

## Conclusion

In conclusion, Langerhans cell histiocytosis is a rare disease. It has a male predominance mainly affecting children. In particularly, LCH often involves the orbit. The lesions of orbital LCH most frequently involved the superior or superlateral orbital roof, lesions location were superficial. Through our research, We found the following characteristic manifestations: most of the lesions showed hyper-hypointense mixed signals on T2WI, showed isointensity and iso-hypotense signals on T1WI. LCH is prone to hemorrhage, necrosis and cystic changes. Some lesions surrounded by a circular low signal. The edges of the lesions and the surrounding tissue presented more significant enhancement than the central area, which has certain specificity to help the diagnosis of LCH. More importantly, these characteristics have not been reported in other literatures. Therefore, MRI findings could provide crucial information for clinicians, and combining with existing research results, MRI characteristic features can improve the diagnostic accuracy of the orbital LCH.

## Supplementary information

**Additional file 1: Table S1** Patients age, sex and duration

## Data Availability

The data supporting the conclusions of this article are contained within the manuscript. The datasets used and/or analyzed during the current study are available from the corresponding author on reasonable request.

## Abbreviations

FS: Fat saturation
FSE: Spin-echo
LCH: Features of orbital Langerhans cell histiocytosis
MRI: Magnetic resonance imaging
T1WI: T1-weighted imaging
T2WI: T2-weighted imaging
PACS: Picture archiving and communicating system
DWI: Diffusion weighted magnetic resonance imaging
HE: Hematoxylin-eosin
IHC: Immunohistochemical
