# Supplementary material for: MR imaging features of orbital Langerhans cell Histiocytosis
Source: BMC Ophthalmol. 2019 Dec 19;19:263. doi: 10.1186/s12886-019-1269-9 (PMC6923963; doi:10.1186/s12886-019-1269-9)
Supplement: Supplementary file 1 — Additional file 1: Table S1 Patients age, sex and duration [file 12886_2019_1269_MOESM1_ESM.doc]

| Case number | Age | Sex | disease duration |
| --- | --- | --- | --- |
| 1 | 3 years | female | 1 month |
| 2 | 21 years | male | 2 months |
| 3 | 2 years | male | 1.5 months |
| 4 | 2 years | female | 1 month |
| 5 | 1 year | male | 1 month |
| 6 | 9 months | male | 2 months |
| 7 | 5 years | male | 1 year |
| 8 | 14 years | male | 1 month |
| 9 | 9 years | male | 1 month |
| 10 | 13 years | male | 1 month |
| 11 | 1 year | male | 6 days |
| 12 | 10 years | female | 2 months |
| 13 | 3.1 years | male | 20 days |
| 14 | 3 years | male | 2 months |
| 15 | 1.5 years | male | 1 month |
| 16 | 3 years | male | 2 years |
| 17 | 4.3 years | male | 2 months |
| 18 | 5.2 years | male | 1 month |
| 19 | 21 years | male | 1 week |
| 20 | 11 years | male | 1 month |
| 21 | 3.5 years | female | 1 month |
| 22 | 4 years | female | 2 week |
| 23 | 1.5 years | male | 20 days |

Additional file 1

Table: Patients age, sex and duration
